# Letter from Colonel Maurice, C.M.G.

**Published:** 1918-07-20

**Authors:** G. T. K. Maurice

**Affiliations:** Colonel, A.M.S. Brighton


### LONDON HOSPITAL NURSES.

### Further Correspondence in "The Times."

Following on the letter from Viscount Knutsford in *The Times* of July 9, which we reprinted in last week's issue, p. 333, the following correspondence has appeared in *The Times*:

### Letter from Colonel Maurice, C.M.G.

Sir,—Lord Knutsford has made the mistake of allowing irritation to goad him to display bad manners in public controversy. A man in his position must know it is not good taste to miscall another's official designation nor to sneer by inverted commas. Major W. A. Chapple, M.P., R.A.M.C., is serving under my command and doing right good work too. I will defend my own man, if you will allow me, and the more readily because I am much interested in nursing affairs. The point raised in Parliament is of definite public interest, and the conduct of Lord Knutsford and the London Hospital is condemned by many, though all acknowledge that the motive of the questionable policy is good and that the proceeds are devoted to the splendid charitable work of the London Hospital; work to which Lord Knutsford has devoted time, energy, ability, and money in unstinted measure.

But no one can disguise the fact that, no matter what advantage their labours gain for them, the nurses of the London Hospital are exploited for the benefit of that charity to the extent of £6,000 a year. It is too large a profit to be rightly made out of them.

Even with the intensive training claimed in Lord Knutsford's letter it is doubtful if a nurse should be properly described as trained after only two years' work, and as a fact the London Hospital does not give a full certificate at the end of two years but at the end of four. After her first two years a nurse has had the amount of training that enables her in her third year to profit exceedingly by the vast and varied experience she can get in a hospital's wards and nowhere else. But at the London, in order to get money for the charity, it seems she is sent out to any case for which she may be requisitioned.

Neither Lord Knutsford nor the most enthusiastic apologists for the London Hospital will contend that the experience gained by a nurse when looking after the ordinary case that requires her services in a private house is equal to the experience to be gained in the wards, or that the instruction given by the medical man in charge of a private case equals the teaching given by skilled matrons and sisters in a hospital. The objections to the course pursued at the London are that a profit is taken on the labours of the nurses that rightly should belong to them, that the instruction of the junior members of the nursing staff is made secondary to earning that profit, and that nurses not in fact fully certificated are passed off on the public without those who pay for them being aware that the nurses they get are not fully certificated. To these objections Lord Knutsford's letter does not reply. The existence of such a state of things, no matter that the object is good, is wrong and makes a strong case for the passing of a Bill to register certificated nurses.—Yours faithfully, G. T. K. Maurice, Colonel, A.M.S.

Brighton, July 9.

